# Multi-Stimuli Responsive Viologen-Imprinted Polyvinyl Alcohol and Tricarboxy Cellulose Nanocomposite Hydrogels

**DOI:** 10.3390/s24216860

**Published:** 2024-10-25

**Authors:** Salhah D. Al-Qahtani, Ghadah M. Al-Senani, Muneera Alrasheedi, Ard elshifa M. E. Mohammed

**Affiliations:** 1 Department of Chemistry, College of Science, Princess Nourah bint Abdulrahman University, P.O. Box 84428, Riyadh 11671, Saudi Arabia; sdalohtany@pnu.edu.sa; 2 Department of Chemistry, College of Science, Qassim University, Buraidah 51452, Saudi Arabia; ar.mohamed@qu.edu.sa

**Keywords:** polyvinyl alcohol, cellulose, viologen polymer, hydrogels, trichromism

## Abstract

Photochromic inks have shown disadvantages, such as poor durability and high cost. Self-healable hydrogels have shown photostability and durability. Herein, a viologen-based covalent polymer was printed onto a paper surface toward the development of a multi-stimuli responsive chromogenic sheet with thermochromic, photochromic, and vapochromic properties. Viologen polymer was created by polymerizing a dialdehyde-based viologen with a hydroxyl-bearing dihydrazide in an acidic aqueous medium. The viologen polymer was well immobilized as a colorimetric agent into a polyvinyl alcohol (PVA)/tricarboxy cellulose (TCC)-based self-healable hydrogel. The viologen/hydrogel nanocomposite films were applied onto a paper surface. The coloration measurements showed that when exposed to ultraviolet light, the orange layer printed on the paper surface switched to green. The photochromic film was used to develop anti-counterfeiting prints using the organic hydrogel composed of a PVA/TCC composite and a viologen polymer. Reversible photochromism with strong photostability was observed when the printed papers were exposed to UV irradiation. A detection limit was monitored in the range of 0.5–300 ppm for NH_3(aq)_. The exposure to heat (70 °C) was found to reversibly initiate a colorimetric change.

## 1. Introduction

There are various documents that have been used to protect secret information, such as passports, banknotes, court papers, and personal identity cards [1,2,3]. The security and authenticity of such documents are of the utmost importance to prevent their counterfeiting. It is possible to identify counterfeiting when someone makes an illegal copy of a product, document, currency, or medication [4,5]. Another kind of document counterfeiting is the addition, deletion, or erasure of text in a particular document. Consequently, it is vital to confirm the authenticity of documents. The forgery of commercial products costs businesses and consumers billions of dollars annually. Furthermore, personal safety might be put at risk due to the usage of counterfeit components in products such as electrical and medical tools [6,7,8]. This has led to an increase in the necessity for advanced and low-cost anti-counterfeiting methods. Commercial products have been authenticated using a variety of indicator components, such as deoxyribonucleic acid, polymers, peptides, and metal emulsions [9]. Verifying the authenticity of a document requires three distinct assessment steps. The initial step of the examination process, which is based on human identification, is to identify document elements such as holograms and watermarks. In the second step of inspection, fluorescent dyes, a barcode scanner, and an ultraviolet light source are used. The next step is to employ high-tech forensic equipment such as spectrophotometers, infrared spectroscopy, and electron microscopes to identify the document details [10,11]. Documents can be authenticated by including an indicator agent into a certain pattern that is adhered to a paper surface. Various chemicals can be included in the paper-printing process to act as marks against other solvents and/or chemicals that are usually used to remove sensitive data. Bleaching has the potential to oxidize marking agents, which can leave behind durable markings that are indicative of a counterfeiting trial [12]. It is possible to include a solvent indicator into a document bulk to avoid dyestuff leakage during solvent bleaching. Printing different patterns and/or agents onto paper can enhance its security and make it more resistant to counterfeiting. In specific security patterns that make use of intaglio prints, 3D printing can be used as a concealed image to expose the legitimacy of documents from extreme angles. Digital scan traps, such as screen angle and dot frequency modulation, have been used in modern pattern techniques [13,14].

Optical encoding of photographs has been used as an anti-counterfeiting method for documents, protecting images that can be presented sequentially upon tilting the document. Ultraviolet-induced photochromic or fluorescent designs have been used as a simple method of authentication. The inclusion of securing components on document surfaces using metallic and bright materials has proven a significant, simple, and practical authentication strategy [15,16]. The use of photoluminescent agents as security agents to protect sensitive data from counterfeiting is possible because they degrade when peeled or scraped. The development of an optical pattern that is simple to copy but hard to replicate has been reported as a novel anti-counterfeiting method. Some examples of optical authentication materials are plasmon nanoparticles and quantum dots. The capacity of a substance to undergo a color change in reaction to lightening conditions is known as photochromism [17,18]. One application of smart photochromic materials is the production of security barcodes from anti-counterfeiting composites. This authentication barcode can change color in a reversible way when exposed to ultraviolet light. Photochromism has been used for the development of various products such as eyeglasses, sensors, intelligent packaging, optical data storage, smart displays, and military camouflage [19,20,21].

Viologens have been the topic of much research in the pursuit of chemical-, pressure-, electrical-, light-, and heat-sensitive materials. Therefore, viologens are promising building blocks for future smart materials such as molecular switches, batteries, sensors, and materials for selective gas adsorption and separation [22,23,24]. Because of their ability to undergo a reversible color change without fatigue when subjected to a REDOX process, viologens have found use as electrochromic tools. For biological redox reactions, they work well as colorimetric agents. They are redox agents that are very reversible, as well as being reasonably inexpensive and very stable in comparison to other organic molecules with redox activity. Thus, viologens can be used as a successful candidate for photochromic authentication as they do not alter the essential characteristics of documents, such as appearance and mechanical performance [25,26,27,28,29]. However, photochromic inks have shown poor durability. Self-healing materials have shown the great ability to self-repair from damage, considerably improving their durability [30,31,32]. Thus, the development of advanced self-healable anti-counterfeiting hydrogel inks is critical for efficient authentication. The self-healing performance of cellulose hydrogels has been significantly recognized for various pharmaceutical products, such as skin care [33,34,35]. Cellulose is distinguished by biocompatibility, renewability, and biodegradability. Polyvinyl alcohol has been widely used to develop biodegradable materials [36,37].

Herein, thermochromic, photochromic, and vapochromic strips were prepared by stamping with a viologen-containing hydrogel. The printed orange layer became green when irradiated with ultraviolet light. The viologen polymer was synthesized by condensing bipyridinium dialdehyde with dihydrazide in an acidic water medium. The synthesized viologen covalent organic polymer is totally water insoluble due to polar substituents (acylhydrazone and hydroxyl) and cationic bipyridinium groups [38]. Thus, it can be described as a good candidate when dispersed in hydrogel ink. Using the combination of a viologen polymer and a PVA/TCC composite, a photochromic authentication hydrogel can be stamped onto a paper surface. The PVA/TCC composite was cross-linked to form a multi-stimuli responsive coating, which effectively trapped the viologen polymer. The findings demonstrated that the generated film is an efficient authentication composite in terms of photochromism. The color variations in a stamped layer were examined using the absorption spectra and coloration parameters. Several analysis methods were employed to inspect the surface morphology, vapochromism, thermochromism, and photochromism of prints. These methods included energy-dispersive X-ray (EDX), scanning electron microscopy (SEM), and infrared spectroscopy (FTIR). The molecular structure of the resulting viologen polymer was investigated by ^1^H NMR. The findings of X-ray diffraction (XRD) and transmission electron microscopy (TEM) showed that nanoscale particles were formed in an aqueous dispersion of the viologen polymer. Additionally, photostability, mechanical performance, and rheology were investigated. With its promising optical features, the current photochromic nanocomposite based on viologen polymers can improve the performance of encoding prints. Tricarboxy cellulose, containing three carboxylic units on the anhydro-glucose moiety, was prepared by the oxidation approach. PVA/TCC hydrogels were developed by the freezing/thawing approach. The viologen-stamped sheets underwent a quick and reversible color change from orange to green upon exposure to ammonia (NH_3_). The photochromic performance was investigated at different contents of the viologen polymer. The morphological and mechanical behaviors of prints were explored, together with the rheological features of the produced composites.

## 2. Experimental

### 2.1. Materials

Chemicals and solvents were obtained from Merck (Darmstadt, Germany). Reaction progress was followed using TLC (F254, Merck, Germany). The synthesized compounds and intermediates were exposed to purification using flash column chromatography employing silica gel 60. Using previously reported methods [38], 5-hydroxyisophthalohydrazide 1 and 1,1′-bis(4-formylbenzyl)-4,4′-bipyridin-1,1′-dium dibromide 2 were synthesized. The sodium periodate (NaIO_4_), sodium bromide (NaBr), Whatman sheets, sodium hypochlorite (NaClO), microcrystalline cellulose (Avicel^®^PH 101; DP 135), (2,2,6,6-tetramethylpiperidin-1-yl)oxyl (TEMPO), and PVA (99%; Mw 8.9–9.8 × 10^4^) were obtained from Sigma-Aldrich (Darmstadt, Germany). TCC was prepared using a previously reported procedure [39].

### 2.2. Preparation of TCC

**Figure 1:**
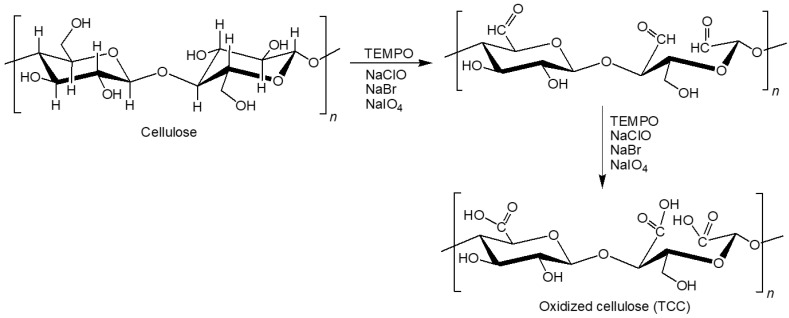
Preparation of tricarboxy cellulose.

A suspension of cellulose (10 g) in distilled water (1200 mL) was inserted into a round-bottom flask (4 L; wrapped with aluminum foil to avoid periodate decomposition). A combination of NaIO_4_ (5.4 g, 45 mmol), NaBr (8 g, 80 mmol), and TEMPO (0.8 g, 5 mmol) was gradually poured into the aforementioned cellulose solution while stirring. NaClO_(aq)_ (9%; 5.9 g, 80 mmol) was poured into the aforementioned combination. The solution provided was stirred for an additional 5 h. The solution pH was maintained at ~10.5 by using NaOH_(aq)_ (2 M). The reaction progress was quenched by adding glycerol (6 mL), filtered under vacuum, and rinsed with distilled water and HCl_(aq)_ (0.5 M). An excessive amount of absolute ethanol (10% *v*/*v*) was poured into the produced solution, and the provided residue was then centrifuged. The generated powder was dispersed in distilled water, subjected to de-salting, and exposed to diafiltration with a polyether sulfone-based ultrafiltration Millipore membrane. TCC was then separated by freeze-drying. The carboxyl content of TCC was determined upon oxidation using potentiometric titration. Figure 1 displays the preparation procedure of tricarboxy cellulose.

### 2.3. Synthesis of Viologen Polymer

**Scheme 1:**
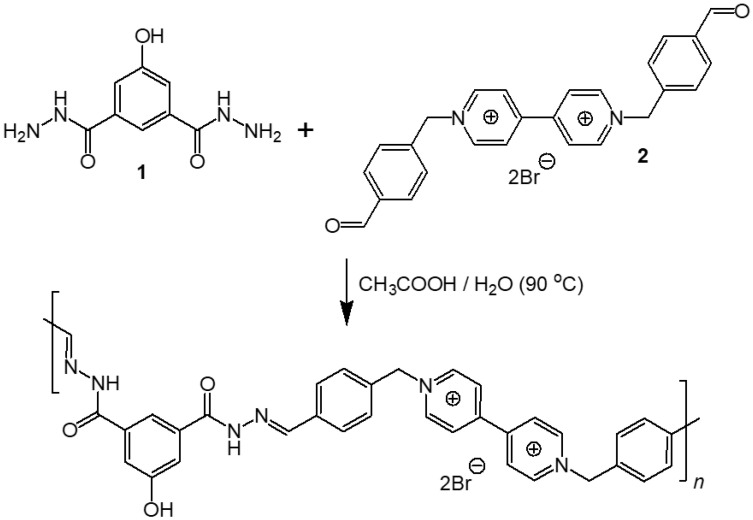
Synthesis process of viologen polymer.

Scheme 1 displays the synthesis procedure of the viologen polymer. A solution of adduct 1 (270 mg, 0.48 mmol) in water (20 mL) was poured into another solution of adduct 2 (101 mg, 0.48 mmol) in a mixture of acetic acid and water (2:10 mL). Following a heating step at 100 °C, the combination was allowed to settle for 20 h under ambient conditions. Gel permeation chromatography (GPC; dimethylformamide): polydispersion index, *M*w, *M*z, *M*_z+1_, *M*p, *M*n, and *M*v were determined at 1.11, 10,500 g/mol, 11,200 g/mol, 11,700 g/mol, 11,500 g/mol, 9200 g/mol, and 10,900 g/mol, respectively. ^1^H NMR (400 MHz, DMSO-d_6_): 12.17 (s, 1H), 10.51 (s, 1H), 9.13 (d, 4H), 9.50 (d, 1H), 9.04 (s, 1H), 8.86 (d, 4H), 8.79 (s, 2H), 8.66 (s, 1H), 8.19 (d, 2H), 8.06 (d, 2H), 7.78 (s, 1H), 7.62 (s, 1H), and 6.14 (m, 1H).

### 2.4. Preparation of Photochromic Hydrogel

An aqueous combination of polyvinyl alcohol/tricarboxy cellulose (85/15) was subjected to three freezing–thawing cycles. In order to accomplish slow thawing to guarantee the creation of a spongy architecture, the solution was placed in a fridge at 5 °C after each freezing cycle. The viologen polymer (VP) was added at various contents (*w*/*v*), including 0% (VP_0_), 0.25% (VP_1_), 0.5% (VP_2_), 0.75% (VP_3_), 1.00% (VP_4_), 1.25% (VP_5_), 1.50% (VP_6_), 1.75% (VP_7_), and 2.00% (VP_8_), to produce various printing pastes. The mixture was further homogenized for 15 min at 25 kHz. A wooden stamp was used as a simple process to apply the prepared hydrogel inks onto sheets of Whatman paper. The stamped papers were air-dried for 30 min. Moreover, the produced viscous solutions were cast in a Petri dish and air-dried for 1 h to produce a film.

### 2.5. Analysis Methods

The NMR analysis was measured using a Bruker Avance 400 MHz Spectrophotometer (Fällanden, Switzerland). To analyze the structural morphology of the printed papers, Quanta SEM FEG-250 (Brno, Czech Republic) was utilized in combination with TEAM-EDX. Using TEM (JEOL-1230, Tokyo, Japan), the sizes of the viologen polymer particles were investigated. In order to examine the functional groups on the paper surface, Nexus-670 (FTIR; Nicolet, USA) was used. The XRD analysis of a milled viologen powder was studied by XRD (PANalytical Pro-X-Pert, Germany). GPC (Agilent PL220, Santa Clara, CA, USA) was used to analyze the viologen powder in DMF. A thermogravimetric analyzer (Perkin-Elmer STA6000) was used to study the thermal behavior of the viologen polymer at a heating rate of 10 °C/min. Using a 100 N load cell attached to a Zwick-Universal, the mechanical behavior of stamped sheets was conducted. Viscosity and rheology were studied using a Brookfield DVIII Rheometer at different shear rates.

### 2.6. Colorimetric Screening

After subjecting the stamped strips to vapochromic, thermochromic, and photochromic experiments, their CIE (Commission Internationale de l’Eclairage) Lab coordinates and color strength (*K/S*) were conducted using an Ultrascan Pro (HunterLab; Reston, VA, USA). The CIE Lab coordinates are represented by a* for the red (+a*) to green (–a*) coordinate, b* for the yellow (+b*) to blue (–b*) coordinate, and L* for the white (100) to black (0) coordinate. The International Commission on Illumination (CIE Lab) is used to express colors as 3D numerical coordinates [9]. In order to examine the sample reversibility, the absorption spectra of the viologen-coated paper sample were taken before and after exposure to ultraviolet light, ammonia vapor, and heat numerous times.

## 3. Results and Discussion

### 3.1. Morphological Studies

**Figure 2:**
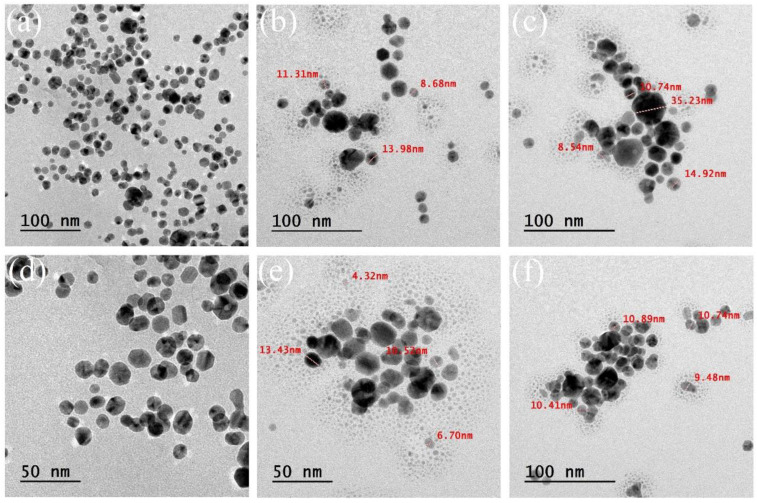
TEM analysis of viologen polymer particles at different magnifications and different positions in the tested sample (**a**–**f**).

Smart materials can change color in reaction to external stimuli, such as heat and light. Photochromic materials can switch color upon exposure to light [20]. The potential of photochromic papers has increased lately, owing to their various potential applications, such as sensors. Figure 2 shows transmission electron microscopy images of the produced covalent organic viologen polymer, displaying particle diameters of 4–35 nm. The nanostructure of the viologen polymer that facilitated its uniform distribution over the document surface was made possible by their modest size.

**Figure 3:**
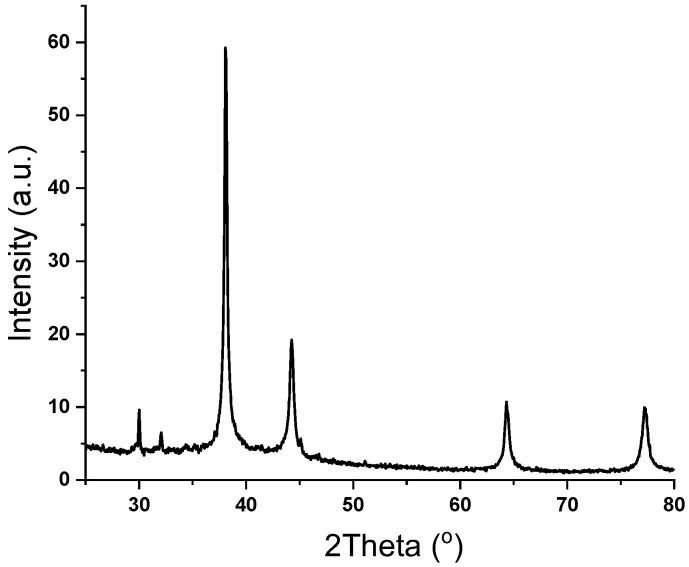
XRD spectrum of polymer nanoparticles.

As shown in Figure 3, the XRD spectrum was utilized to study the crystal structure of the viologen particles. As a result of d-spacing, a strong band was detected at 2θ = 38.04°. This suggests that the material is mostly amorphous and encloses many π-stacking attraction forces. Two signals were identified at 2θ = 44.18°, 64.36°, and 77.25°, which are probably indicators of bromide-based ion-paired counterions. The typical absorption peak of the asymmetric stretching vibration for the TCC carboxyl was determined at 1721 cm^−1^ [40].

**Figure 4:**
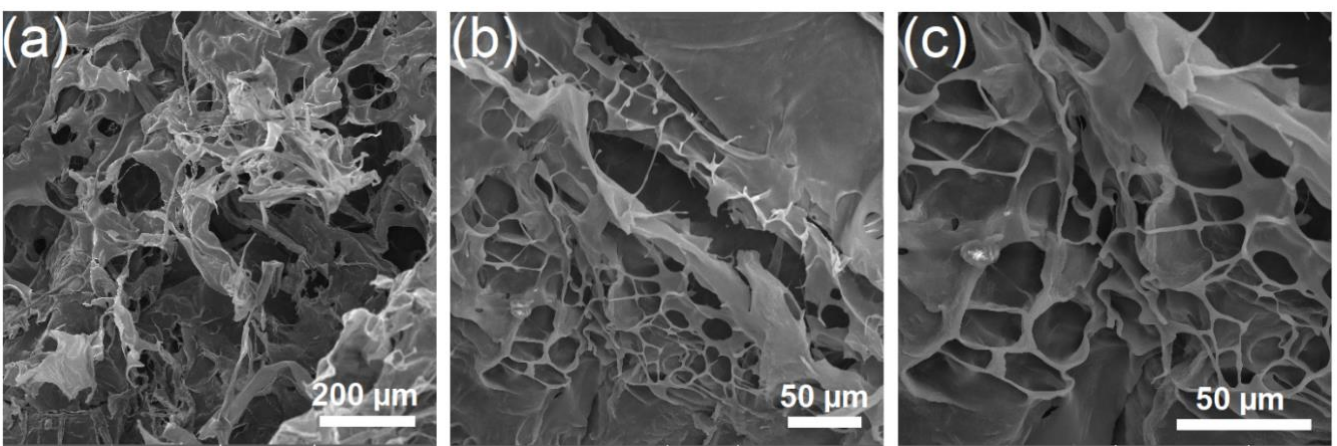
SEM analysis of cast hydrogel film; VP_6_ (**a**–**c**).

**Figure 5:**
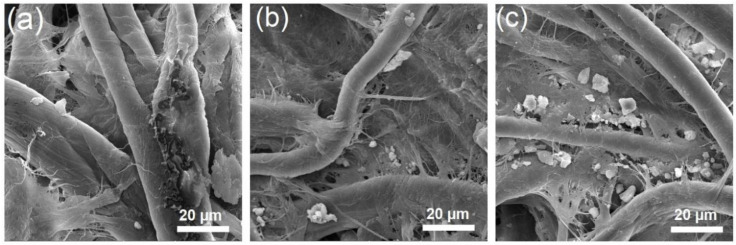
SEM images of printed paper (VP_6_) at different positions on the sample surface (**a**–**c**).

The use of viologen nanoparticles enhances their dispersion in the hydrogel layer printed onto paper surface. The multi-stimuli responsive hydrogel was printed onto a paper surface by using a wood stamp as an easy and inexpensive technique [41]. Figure 4 shows SEM images of cast hydrogel film immobilized with the viologen nanoparticles, demonstrating a porous aerogel structure. Figure 5 shows the SEM images of the multi-stimuli responsive printed paper sheets. SEM images showed that the viologen polymer nanoparticles were successfully coated onto a paper surface. The printing process effectively and uniformly distributed the viologen polymer nanoparticles onto the document surface, as shown in the microscopic images. One possible explanation for the homogeneous dispersion of the viologen polymer nanoparticles could be the formation of hydrogen bonding between polymer nanoparticles and hydroxyl oxygen lone pairs on cellulose paper. Although the viologen particles were dispersed throughout the paper surface, the paper cellulose microfibrous morphologies of the blank paper and the stamped sheets were identical. According to EDX analysis (Table 1), the hydrogel coating on the document surface showed homogeneous distribution of the viologen polymer. The cellulosic paper sheets and the PVA/TCC composite were found to be mostly composed of oxygen and carbon, whereas the viologen polymer nanoparticles consisted of nitrogen and bromine in trace quantities. In order to study the thermal characteristics of the viologen polymer, thermogravimetric analysis (TGA) was used. Its three thermal phases were indicated by its thermal stability up to 275 °C. Due to moisture evaporation, the first phase was detected in a temperature range of 25–190 °C. With a weight loss of almost 40% owing to the thermal degradation of polymer, the second phase was determined at a temperature range of 190–340 °C. The third phase was determined at 340–400 °C [42,43].

EDX analysis determined the elemental contents and efficiency of the viologen dispersion on the stamped papers, as shown in Table 1. The homogeneous dispersion of the viologen particles on paper was confirmed by the quite similar elemental compositions at the three test sites for the same sample. In order to verify the self-healing property, two hydrogel rectangles of VP_6_ with a relative humidity of 95% were brought into contact with each other. The two rectangle pieces were found to fuse into a single piece able to withstand 100 g. This could be attributed to the H-bonding between the carboxylic group (acceptor) on TCC and the hydroxylic group (donor) on PVA [44,45]. The binding mechanism of the multi-stimuli responsive hydrogel to the cellulose sheet was determined by Fourier transform infrared spectroscopy (Figures S1–S4). The cellulose hydroxyl substituent was monitored at 3348 cm^−1^, whereas the aliphatic group was determined at 2927 cm^−1^. The carbonyl ester of PVA/TCC displays a peak at 1709 cm^−1^ [39,40]. Due to the trace amount of the viologen nanoparticles and the low thickness of the printed layer, no significant shifts were detected in the absorption bands as compared to the blank paper sheet. However, the intensities of the carboxyl and hydroxyl absorption bands were found to decrease after printing, which is explained by the creation of H-bonding between the hydroxyl of PVA/TCC composite and the surface of the paper cellulose [45].

### 3.2. Photochromic Analysis

As shown in Table 2, the coated papers exhibited an orange hue similar to that of the control sample (VP_0_). Slight variations in the colorimetric parameters of the paper sheets were reported after coating. When the viologen content was raised from VP_1_ to VP_8_, both CIE Lab and *K/S* showed significant changes, signifying the formation of a deep orange hue. Similarly, when the viologen content was increased from VP_1_ to VP_8_, significant differences were shown in the CIE Lab and *K/S* when exposed to UV light, signifying the development of a deep greenish hue. L* was found to be lowered with exposure to ultraviolet light, suggesting a color alteration from orange to green. Both of the high +b* and the low –a* values rose in response to exposure to ultraviolet light. It was shown that the color change from orange to greenish was verified with raising –a* and lowering +b*. The findings showed that the green absorption intensity increased as the viologen ratio increased; however, this intensity remained constant at a viologen content greater than VP_6_. Thus, VP_6_ exhibited the highest photochromic absorbance intensity as compared to samples with both lower and higher viologen concentrations, as shown by a weaker or similar green absorbance band, respectively.

The photochromic nanocomposite was built using viologen nanoparticles at different concentrations. The viologen organic polymer was trapped inside the PVA/TCC composite. The PVA/TCC hosting agent that contains viologen nanoparticles was cross-linked on the surface of the paper [39]. Reversible photochromism was observed in the coated samples from 430 nm (orange) to 598 nm (green). A higher concentration of the viologen nanoparticles resulted in a more pronounced absorption band. The paper-coated film, which had an orange color (430 nm), became greenish under ultraviolet light. When irradiated with an ultraviolet lamp, all of the coated samples exhibited rapid, reversible photo-switching as well as emissive properties.

**Figure 6:**
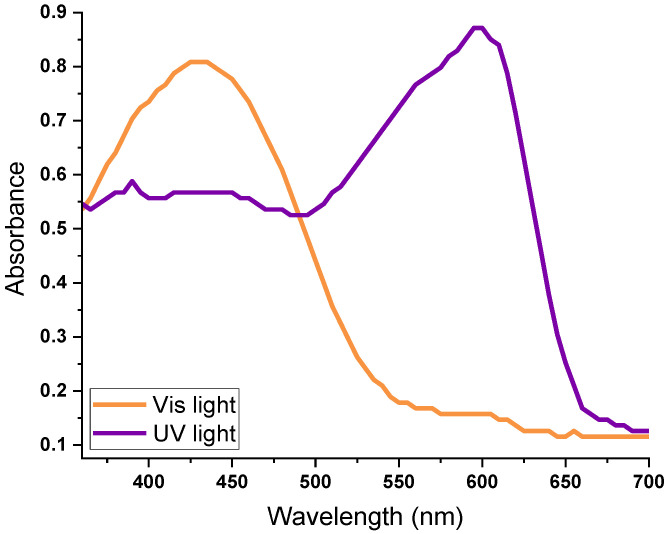
Absorption spectra of VP_6_ below visible (Vis) and ultraviolet (UV) irradiation.

**Figure 7:**
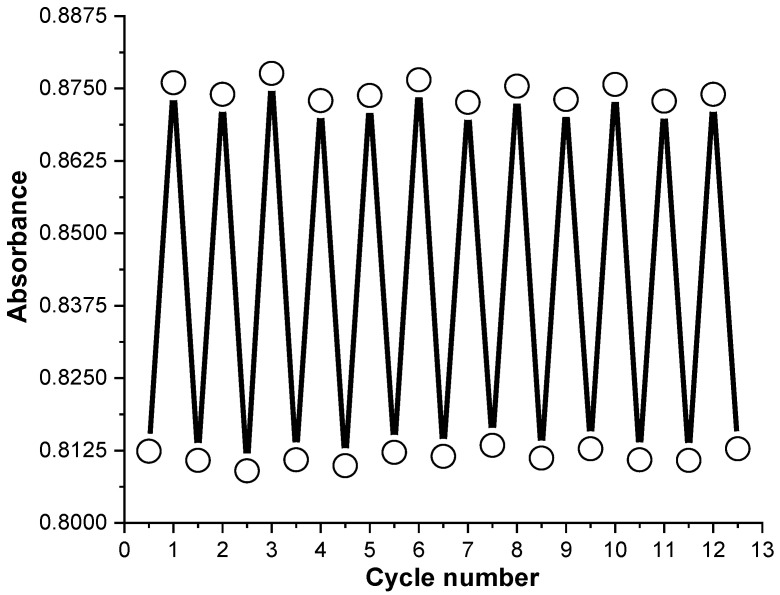
Reversibility of absorbance of VP_6_ over numerous cycles of ultraviolet (598 nm) and visible (430 nm) illumination.

The photochromic performance of prints was investigated as shown in Figure 6. There was no absorbance band detected between 300 and 700 nm for the native paper (VP_0_). A faint absorption peak was identified at 430 nm, which corresponds to the printed orange hue. After ultraviolet irradiation, the light supply was switched off, and the temporal evolution of the absorbance band at 598 nm was studied. The fact that the absorbance peak at 598 nm returned to its pre-irradiated condition after ultraviolet irradiation indicates excellent reversibility without fatigue. An electron transference from a counterion to viologen produces a radical cation due to the electron-deficient nature of the viologen cation. The intramolecular charge transfer between N^0^ and N^+^ causes a bright color in the radical cation state. In addition, it was shown that the absorbance intensity at 598 nm rose when the irradiation duration was extended, probably because more radical cation species were accumulated [38]. The resistance to fatigue was tested by repeatedly exposing the sample to ultraviolet light for 100 s and then putting it in a dark room. The reversible color shift can be repeated numerous times without fatigue (Figure 7). Thus, the current printed paper sheets can be described as a perfect choice for anti-counterfeiting applications owing to their interesting photochromic characteristic.

### 3.3. Mechanical and Rheological Studies

**Figure 8:**
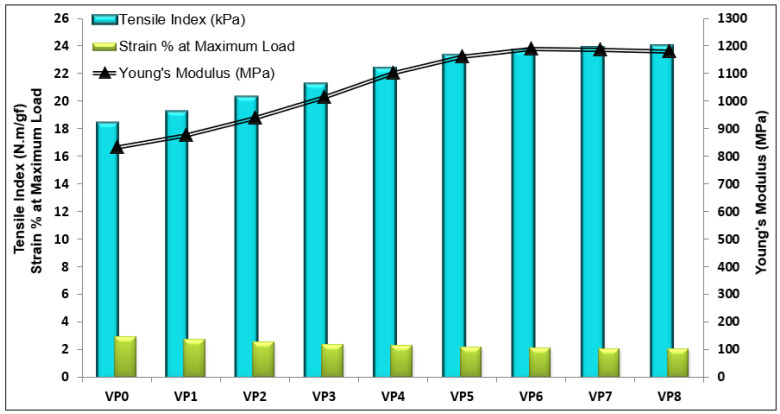
Effect of viologen content on mechanical performance of stamped samples, including tensile strength, strain, and Young’s modulus.

The mechanical properties were examined by covering the paper sample entirely with the produced hydrogel. An increase in tensile index relative to the uncoated sheet (VP_B_) was observed when the viologen-free composite fluid (VP_0_) was applied to the paper sample. The hydrogel (VP_0_), typically composed of a polyvinyl alcohol (PVA)/tricarboxy cellulose (TCC) binder, was accountable for producing the monitored strain percentage. The strain percentage for VP_0_ was higher than that of the VP_1_ sample. Young’s modulus of VP_0_ was found to be almost equivalent to the strain percentage. This can be attributed to the fact that Young’s modulus is tensile- and strain-dependent [46]. One of the important components of the hydrogel ink is the polyvinyl alcohol (PVA)/tricarboxy cellulose (TCC) binder that is responsible for the adhesion and flexibility of VP_0_. The impact of increasing the viologen nanoparticle concentrations on the mechanical properties of stamped papers is illustrated in Figure 8. An increase in the viologen content from VP_1_ to VP_6_ led to an improvement in both Young’s modulus and tensile strength. The coated samples maintained a value close to the initial value while the viologen nanoparticle ratio increased from VP_6_ to VP_8_. The strain percentage at break showed slight changes as the viologen content rose from VP_1_ to VP_8_. An interfacial cross-linking occurred between the viologen polymer and the paper cellulose hydroxyl. As a result of the viologen particles and the paper cellulose coordinatingly binding, the cross-linking among cellulose microfibers was enhanced when the viologen nanoparticles were deposited onto the paper surface. This could be attributed to the formation of hydrogen bonds between the carboxylic groups (acceptors) on TCC and the hydroxylic groups (donors) on PVA [44,45]. The tensile improved up to VP_6_ as the viologen content was increased. Because viologen nanoparticle coagulation expands the interfibrous gaps, the tensile strength was unaffected by VP_6_ and VP_8_. The hydrogel stamping allowed nanocomposite inks to be applied onto the paper sheets, creating an anti-counterfeit document with the smoothest conceivable surface while maintaining its pliability.

**Figure 9:**
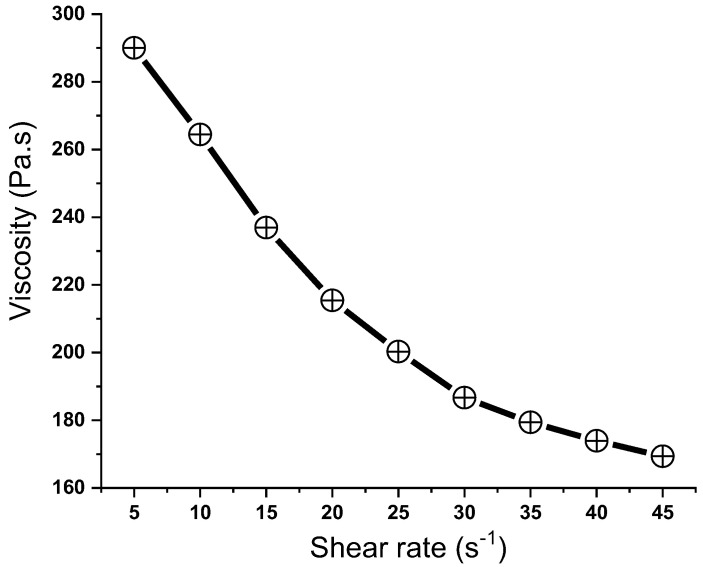
Effect of shearing rate on the viscosity of VP_6_.

Figure 9 shows the flow profile that was utilized to examine the rheological performance of the fluid hydrogel sample (VP_6_). The synthetic nanocomposite ink was thin because its flow curve was carefully controlled to adhere to Newton’s laws. A straight-line reduction in viscosity was achieved by increasing the external shear. The connection between shear rate and viscosity is co-linear. The viscosity of the fluid was shown to quickly drop, nearing equilibrium, at both the low and high shearing rates. Thus, the viscosity of the fluid was unchangeable after increasing the shear rate higher than 45 s^−1^.

### 3.4. Thermochromic Analysis

**Figure 10:**
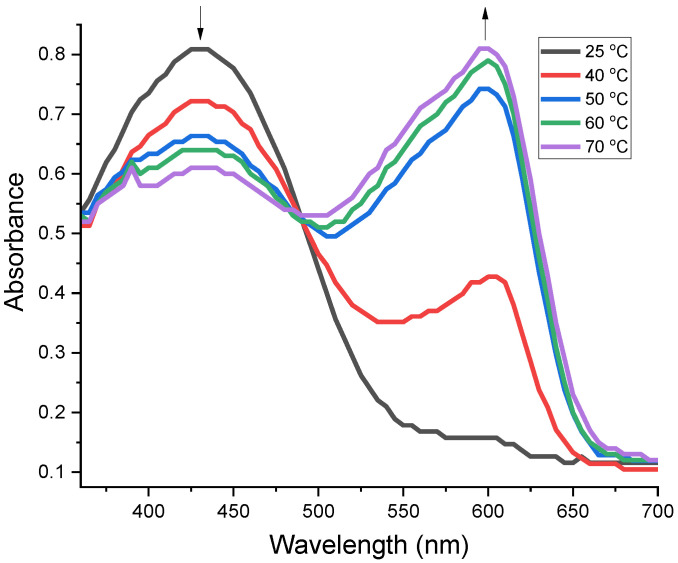
Absorption spectra of VP_6_ at different temperatures.

**Figure 11:**
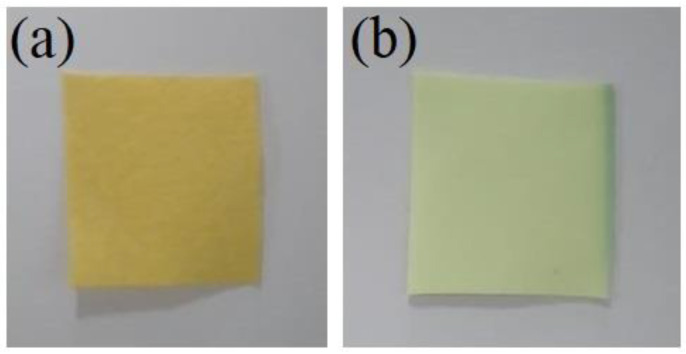
Thermochromism of VP_6_ showing a change in color from orange (**a**) to greenish (**b**) with heating from 25 °C to 70 °C, respectively.

The creation of the equivalent green radical cations can be induced by charge transfer between viologen cations and their counterions when exposed to heat [47]. Figure 10 shows the thermochromic properties of the viologen-stamped paper. Table 3 shows the results of studying the CIE Lab and absorption spectra of VP_6_, which were used to explain the thermochromism of the viologen-stamped paper. Upon raising the temperature from 25 °C to 70 °C, significant variations were observed in the coloration parameters, verifying the creation of a green hue (Figure 11). A colorimetric change from orange to greenish was shown by a reduction in the value of L* as the temperature increased. The high +b* and low –a* increased with raising the temperature from 25 °C to 70 °C. The viologen-stamped paper was tested using various heat/cool cycles to determine its reversibility against heating.

### 3.5. Vapochromic Performance

**Figure 12:**
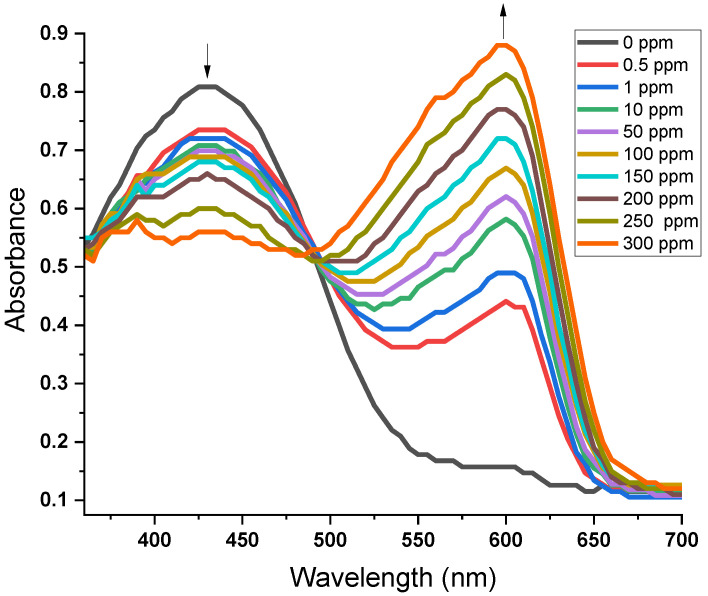
Absorbance spectra of paper (VP_6_) under air and different concentrations of NH_3(g)_.

It has been an important and difficult process to produce colorimetric sensor materials that can detect ammonia gas in both its liquid and gaseous forms by tracking a change in color [48,49,50]. In order to facilitate electron transfer from NH_3(g)_ to viologen, it has been suggested that viologen functions as a Lewis acid while gaseous ammonia functions as a Lewis base. This triggers the color shift by the generation of the viologen radical cation. VP_6_ can be used to monitor ammonia vapor by the naked eye. Excellent sensitivity is achieved by the fast diffusion of analytes into the micro-scale fibrous cellulose, which has a large surface area and can quickly absorb both gaseous and aqueous ammonia [51]. Figure 12 shows that after passing ammonia vapor onto VP_6_, the color instantly changed from orange to green. This change corresponds to the formation of a prominent absorption band at 598 nm. The green color of the coated paper test strip can be promptly reversed to orange by protecting the paper sample from ammonia gas. The paper strip will once again become green when exposed to ammonia in the same way. By monitoring the absorbance at 598 nm for numerous cycles of exposure to ammonia vapor, the resistance to fatigue of the stamped sheet to ammonia was evaluated. Reversible color changes were shown to be repeatable for numerous cycles without fatigue. The present ammonia sensor has many possible uses, such as environmental protection, human health monitoring, and monitoring of industrial operations.

**Figure 13:**
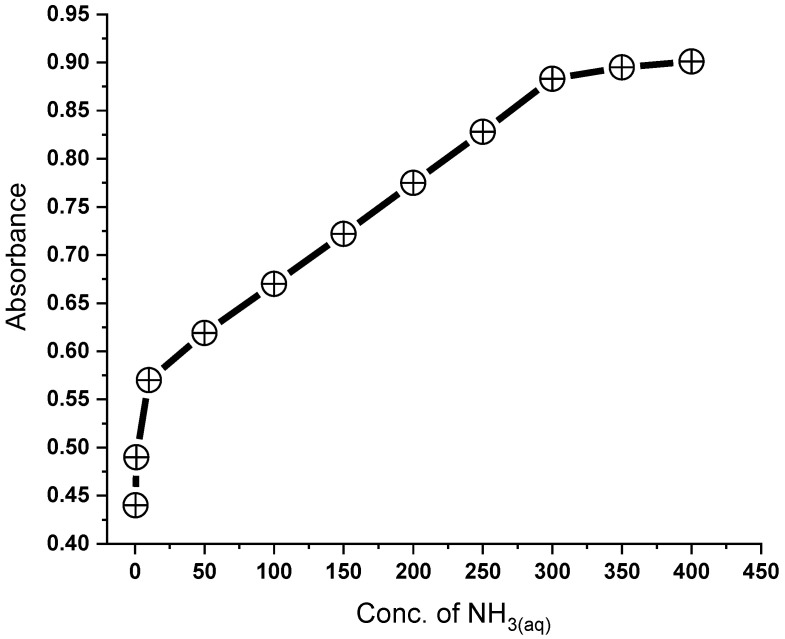
Absorption intensity of VP_6_ versus various concentrations of NH_3(aq)_.

Viologens are electron-deficient agents with a high ability to perform an intermolecular electron transfer between donor and acceptor units, resulting in colorimetric changes. Thus, they have been employed as a multifunctional responsive molecular unit to play a key role in chromic materials. This color change is attributed to the intermolecular electron transfer from ammonia (electron donor) to viologen (electron acceptor) [22,23,24,25,26,27,28]. In order to investigate the aqueous ammonia detection limit, the viologen-stamped paper was submerged in solutions containing varying contents of ammonia for durations ranging from 1 to 5 s. After letting the viologen-stamped strip air-dry, the colorimetric changes in the absorbance wavelength intensity were measured. As shown in Figure 13, the absorption wavelength of VP_6_ rises from 430 nm to 598 nm when the content of NH_3(aq)_ increases from 0.5 ppm to 300 ppm. At an ammonia content of 0.5 ppm, the absorbance band at 598 nm became visible. Intense absorption at 598 nm is proportional to the quantity of ammonia in water. At ammonia contents greater than 300 ppm, however, no changes in absorption intensity were detected.

## 4. Conclusions

In summary, multichromic paper sheets that are highly reversible showed a color change from orange to green when exposed to gaseous ammonia, ultraviolet light, and heat up to 70 °C. A smart hydrogel ink immobilized with a viologen polymer was printed onto the paper sheets. An acidic aqueous solution was used to synthesize viologen polymer by a simple polymerization process. Viologen nanoparticles helped assemble authenticating nanocomposite films that were useful for anti-counterfeiting purposes. In order to develop hydrogel inks, solutions of polyvinyl alcohol and tricarboxy cellulose (self-healable composite) and different contents of viologen nanoparticles (chromic agent) were prepared. The polyvinyl alcohol/tricarboxy cellulose composite was used as a trapping matrix to adhere the viologen nanoparticles to the surface of the paper. The viologen-stamped paper was exposed to variable contents of aqueous ammonia to demonstrate a detection limit of 0.5–300 ppm. The current strategy can be employed to afford a simple, inexpensive, and durable anti-counterfeiting coating. Two absorbance peaks were detected at 430 and 598 nm for the printed paper sheets. When dispersed in water, the viologen particles had a diameter ranging from 4 to 35 nm. A variety of methods were used to analyze the coated paper sheets, coloration parameters, and absorption spectra. The orange hue of the printed paper became greenish when exposed to an ultraviolet source.

## Figures and Tables

**Table 1:** Elemental contents of VP_0_, VP_1_, and VP_8_ at some selected sites on paper surface.

Sample		C	O	Br	N
VP_0_		55.26	45.74	–	–
VP_1_	Site 1	55.40	44.57	0.50	0.53
	Site 2	55.64	44.32	0.43	0.61
	Site 3	55.13	44.87	0.42	0.58
VP_8_	Site 1	55.26	44.07	0.84	0.83
	Site 2	55.30	44.02	0.75	0.93
	Site 3	54.89	44.47	0.74	0.86

**Table 2:** Colorimetric screening of prints underneath visible (*A*_1_) and ultraviolet (*A*_2_) lights.

Sample	L*		a*		b*		*K/S*	
	*A*_1_	*A*_2_	*A*_1_	*A*_2_	*A*_1_	*A*_2_	*A*_1_	*A*_2_
VP_0_	86.40	83.49	2.23	2.78	1.42	1.77	0.37	0.40
VP_1_	85.96	77.10	–3.23	–6.52	9.59	3.96	0.66	3.09
VP_2_	85.53	76.59	–2.90	–7.08	10.50	3.45	0.87	3.43
VP_3_	84.87	75.48	–2.48	–7.52	11.00	2.82	0.93	3.80
VP_4_	84.06	73.96	–2.20	–8.43	12.82	2.62	1.09	4.15
VP_5_	83.81	73.41	–1.97	–9.67	14.68	2.21	1.16	4.40
VP_6_	83.42	71.09	–1.65	–10.86	15.25	2.08	1.39	4.76
VP_7_	82.86	70.75	–1.39	–12.15	16.44	1.73	1.41	5.14
VP_8_	82.37	69.38	–1.12	–12.74	16.64	0.96	1.56	5.45

**Table 3:** Color screening of VP_6_ at various temperatures (T).

T (°C)	L*	a*	b*	*K/S*
25	83.42	–1.65	15.25	1.39
40	80.7	−4.63	10.9	2.81
50	77.63	−6.95	8.21	3.52
60	72.55	−9.64	6.77	3.65
70	69.12	−14.79	5.26	4.39

## Data Availability

The data that support the findings of this study are available from the corresponding author upon reasonable request.

## Supplementary Materials

The following supporting information can be downloaded at: https://www.mdpi.com/article/10.3390/s24216860/s1, Figure S1: FTIR spectrum of printed sheet (VP_0_); Figure S2: FTIR spectrum of printed sheet (VP_1_); Figure S3: FTIR spectrum of printed sheet (VP_6_); Figure S4: FTIR spectrum of printed sheet (VP_8_).
