# Supplementary material for: Multi-Stimuli Responsive Viologen-Imprinted Polyvinyl Alcohol and Tricarboxy Cellulose Nanocomposite Hydrogels
Source: Sensors (Basel). 2024 Oct 25;24(21):6860. doi: 10.3390/s24216860 (PMC11548534; doi:10.3390/s24216860)
Supplement: Supplementary file 1 [file sensors-24-06860-s001.zip › sensors-3234893-supplementary.pdf]

Supplementary Data

Multi-Stimuli Responsive Viologen-Imprinted Polyvinyl Alcohol and Tricarboxy Cellulose Nanocomposite Hydrogels

Salhah D. Al-Qahtani [1], Ghadah M. Al-Senani [1,*], Muneera Alrasheedi [2,*], Ard elshifa M. E. Mohammed [2]

[1] Department of Chemistry, College of Science, Princess Nourah bint Abdulrahman University, P.O. Box 84428, Riyadh 11671, Saudi Arabia

[2] Department of Chemistry, College of Science, Qassim University, Buraidah 51452, Saudi Arabia

* Correspondence: gmalsnany@pnu.edu.sa (G.M.A.-S.); mu.alrasheedi@qu.edu.sa (M.A.)

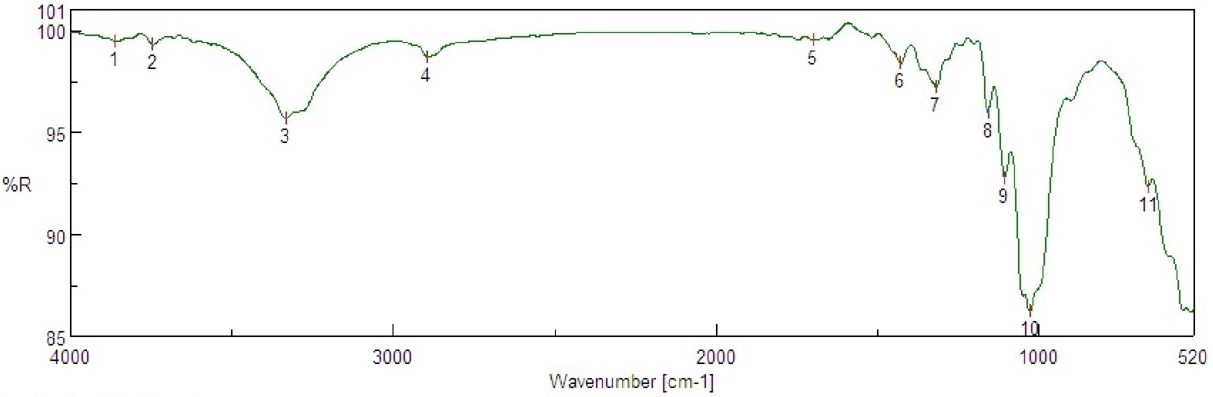

Figure S1. FTIR spectrum of printed sheet (VP_0).

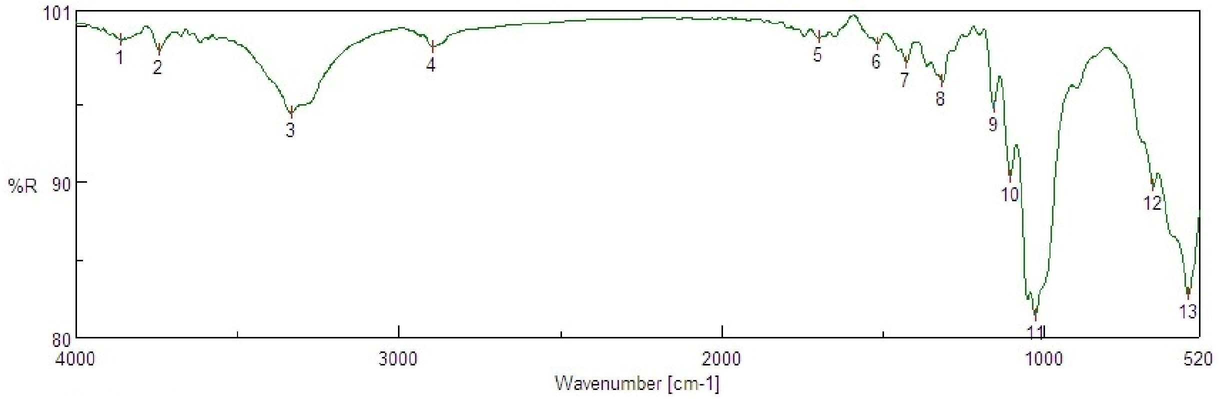

Figure S2. FTIR spectrum of printed sheet (VP_1).

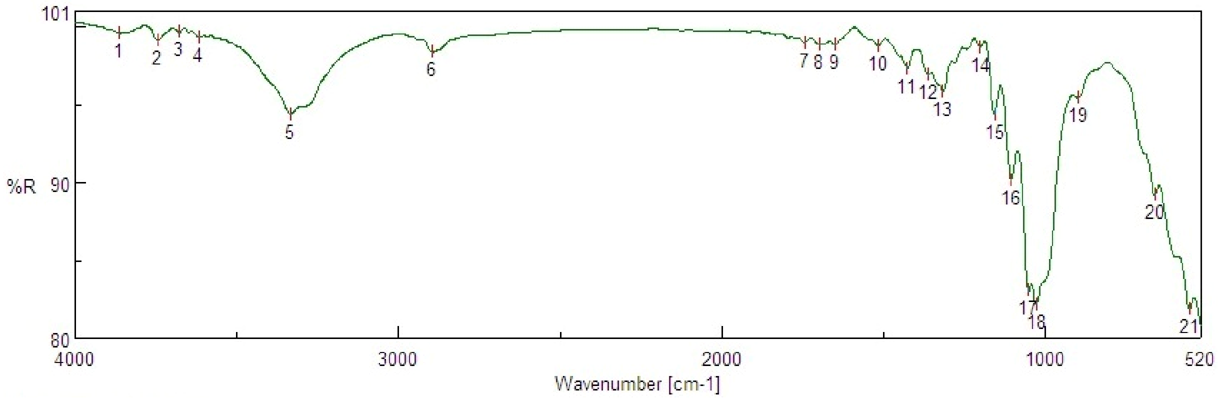

Figure S3. FTIR spectrum of printed sheet (VP_6).

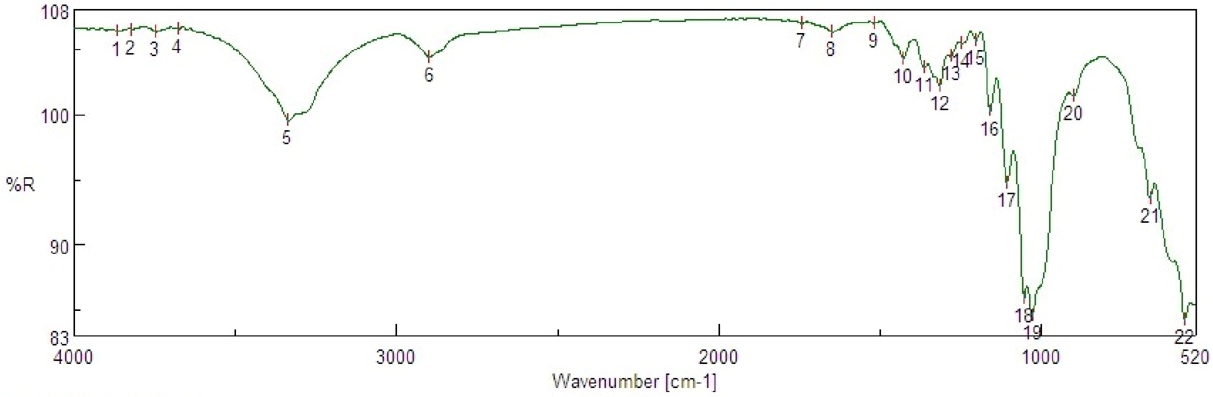

Figure S4. FTIR spectrum of printed sheet (VP_8).
